# Chemical and physical properties of some saline lakes in Alberta and Saskatchewan

**DOI:** 10.1186/1746-1448-4-3

**Published:** 2008-04-22

**Authors:** Jeff S Bowman, Julian P Sachs

**Affiliations:** 1 School of Oceanography, University of Washington, Seattle 98195-5351, USA

## Abstract

**Background:**

The Northern Great Plains of Canada are home to numerous permanent and ephemeral athalassohaline lakes. These lakes display a wide range of ion compositions, salinities, stratification patterns, and ecosystems. Many of these lakes are ecologically and economically significant to the Great Plains Region. A survey of the physical characteristics and chemistry of 19 lakes was carried out to assess their suitability for testing new tools for determining past salinity from the sediment record.

**Results:**

Data on total dissolved solids (TDS), specific conductivity, temperature, dissolved oxygen (DO), and pH were measured in June, 2007. A comparison of these data with past measurements indicates that salinity is declining at Little Manitou and Big Quill Lakes in the province of Saskatchewan. However salinity is rising at other lakes in the region, including Redberry and Manito Lakes.

**Conclusion:**

The wide range of salinities found across a small geographic area makes the Canadian saline lakes region ideal for testing salinity proxies. A nonlinear increase in salinity at Redberry Lake is likely influenced by its morphometry. This acceleration has ecological implications for the migratory bird species found within the Redberry Important Bird Area.

## Background

Canada's Northern Great Plains contain a region of athalassohaline (of a different ionic composition than seawater) lakes extending from Manitoba, across the southern half of Saskatchewan, and into Alberta. This region includes both permanent and ephemeral bodies of water (playas) and displays a range of total dissolved solids from near 0 to 370 g L^-1 ^[1]. These lakes and playas can be categorized by specific conductivity as fresh (less than 80 μs cm^-1^), oligosaline (800–8,000 μs cm^-1^), mesosaline (8,000–30,000 μs cm^-1^), polysaline (30,000–45,000 μs cm^-1^), eusaline (45,000–60,000 μs cm^-1^), and hypersaline (greater than 60,000 μs cm^-1^) as described by Cowardin [2,3] and LaBaugh [3]. A survey of some of the lakes in this system was completed by Hammer [1,4] who compiled detailed information on ion composition and began a time series for several lakes that indicated a secular increase in salinity during the last century. Hammer also presented seasonal variations of TDS in five saline lakes, highlighting the strong connection between seasonal water budgets, water projects, and salinity in these basins. The geochemical nature of the lakes is well described in a review by Last and Ginn [5], who investigated the wide variation in sediment composition between lakes of the Western Great Plains of Canada and attributed those differences to underlying geological processes.

The importance of Canada's saline lakes from an environmental and economic standpoint cannot be understated. Many of the larger saline lakes are critical habitat for migratory birds [6-9]. Redberry Lake itself is designated as a UN Biosphere Reserve [10]. Numerous lakes in the system play a role in Saskatchewan's mineral industry including Big Quill (potassium sulfate production), Chaplin (sodium sulfate extraction), and Ingebright (past sodium sulfate extraction). Other lakes, such as Patience, are greatly affected by mineral extraction [11]. Untapped sodium sulfate deposits exist under several lakes including Muskiki, Little Manitou, and Bitter Lakes [12]. Big Quill is also home to a fishery for *Diaptomus *and *Artemia*, both of which are sold as aquarium food.

A considerable amount of research has been done on phytoplankton and primary production in Canadian saline lakes [13-17]. However relatively little data is available on the diversity of prokaryotic life in these lakes. Although extensive research has been conducted throughout the world on the microbiology of evaporative hypersaline environments, these environments are typically thalassohaline. Where well studied lakes are athalassohaline they tend to be "soda lakes"; highly alkaline lakes containing a high concentration of carbonate [18,19]. Recently Grasby and Londry [20] reported on the microbiology of spring-fed saline lake systems in Manitoba. Sørensen et. al [21], Sorokin et. al [22], and others have described the microbial community structure of hypersaline lakes with high concentrations of chloride and sulfate, a characteristic shared with many Canadian saline lakes. However from an ionic and microbiological standpoint the lakes of Saskatchewan and Alberta represent a unique environment, with sulfate the dominant anion over chloride in most lakes.

Regardless of their ionic composition saline lakes support communities of phototrophic, chemoautotrophic, and heterotrophic microorganisms that in turn support a community of multicellular organisms [20,23-25]. In the most saline lakes multicellular life is limited to the brine shrimp *Artemia *sp. [5,26,27]. The invertebrate communities of saline lakes play a critical role in the ecology of the Great Plains by supporting large populations of migratory birds nesting locally and enroute to and from the high Arctic and Asia [6,8,9,28-30]. Redberry, Big Quill, and the Chaplin Lakes in particular are noted for their importance to migratory bird populations.

The wide range of salinities represented among the lakes of Canada's Great Plains makes them ideal for testing the biochemical responses of prokaryotic and eukaryotic microorganisms to changes in salinity. These responses can ultimately be exploited for the reconstruction of salinity in the geological record [31]. Calibrating such biochemical responses requires accurate data on salinity and water chemistry. The purpose of this study is to identify suitable, accessible lakes across a wide salinity range for comparing biochemical responses to salinity. In addition we will augment the extensive geological data compiled by Last and Ginn [5] with a snapshot of water column data including TDS, dissolved oxygen (DO), pH, specific conductivity, and sediment characteristics. These parameters are known to change dramatically on a seasonal and annual basis. Some of these changes can be observed by using these data to extend the time series begun by Hammer for Little Manitou, Big Quill, Manito, and Redberry Lakes [32].

**Figure 1:**
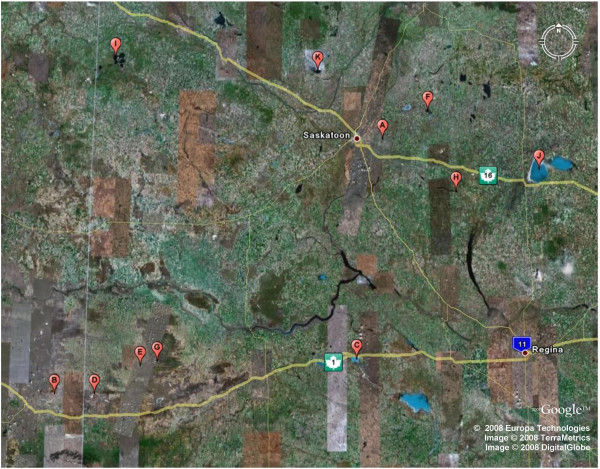
**Locations of lakes investigated**. A. Patience B. Chappice C. Chaplin D. Bitter E. Ingebright F. Muskiki G. Freefight H. Little Manitou I. Manito J. Big Quill K. Redberry. Printed with the permission of Google Earth.

**Figure 2:**
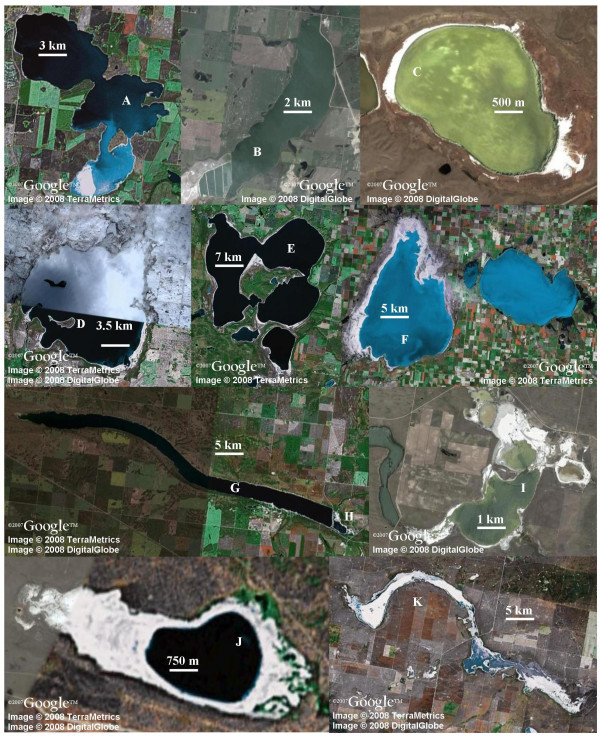
**Satellite images of lakes investigated**. A. Muskiki B. Patience C. Chappice D. Redberry E. Manito F. Big Quill G. Little Manitou West H. Little Manitou East I. Ingebright J. Freefight K. Bitter. Printed with the permission of Google Earth. All images are oriented with north at the top of the image.

A total of 19 lakes were investigated across eastern Alberta and Saskatchewan from June 9–15, 2007. The location where data was collected, specific conductivity, TDS, and absolute salinity (in ppt) for each lake are shown in Table 1. Locations relative to the geography of the region are shown in Fig. 1. Fig. 2 shows satellite images of all lakes investigated and is printed with the permission of Google Earth.

**Table 1:** Sampling location, average specific conductivity (from all measured depths), and TDS for lakes investigated.

Lake	Sample Date	Latitude (degrees)	Longitude (degrees)	Average Specific Conductivity (mS cm^-1 ^at 25°C)	TDS (g L^-1^), depth (m)	Absolute Salinity (ppt)
Patience	6/10/2007	52.1354	106.33	201.35	161.16, 0	139.5
Chappice	6/15/2007	50.13328	110.3698	110.90	193.58, .1	163.6
West Chaplin West Division 1	6/13/2007	50.43772	106.6845	108.50	235.74, .1	183.7
Bitter	6/15/2007	50.13317	109.8707	106.20		
West Chaplin Center Division	6/13/2007	50.43772	106.6845	99.00	192.39, 0	154.8
Ingebright	6/15/2007	50.36329	109.3169	97.36	251.80, .1	160.0
West Chaplin Lake SE Division	6/13/2007	50.41832	106.6735	89.08	144.82, .1	125.7
West Half Chaplin Lake NE Division	6/13/2007	50.43968	106.6458	84.75		
Muskiki	6/11/2007	52.35197	105.7771	57.93	121.47, .1	91.9
Freefight	6/14/2007	50.39685	109.1149	57.71	100.98, 3	94.5
Little Manitou West	6/10/2007	51.71965	105.3944	56.40	85.14, 0	73.7
East Half West Chaplin Lake NE Division	6/13/2007	50.43978	106.641	47.35	60.14, 0	54.1
West Chaplin West Division 2	6/13/2007	50.41922	106.7065	46.62	161.93, 0	137.4
Little Manitou East	6/10/2007	51.71957	105.3944	46.28		
Manito	6/9/2007	52.79052	109.7864	38.58	38.50, .5	38.0
Big Quill	6/11/2007	51.78784	104.3234	20.32	24.87, 0	22.9
Redberry	6/9/2007	52.70732	107.2082	18.72	44.52, 0	40.3
Hughes Bay	6/13/2007	50.40552	106.6555	15.6		
Midtskogen	6/13/2007	50.40552	106.6555	8.35		

### Patience

Patience Lake is a shallow, permanent, hypersaline lake east of Saskatoon in an area of extensive potash extraction. It is unusual as a NaCl system in a region dominated by Na_2_SO_4 _systems. This was first noted by Hammer [1,11], who cited the dumping of potash mine tailings in the lake as the reason for this abnormality. Measurements were taken from a boat near the north end of the lake (specific location given in Table 1). The lake was well mixed and oxygenated throughout the water column. DO ranged from 4.89 mg L^-1 ^at the surface to 4.57 mg L^-1 ^at 2 m, the maximum depth sounded. The pH also stayed constant with depth, from 8.75 at the surface to 8.76 at 2 m. Water at Patience was turbid; the Secchi depth was measured at .3 m. Water samples taken here began to precipitate solids of unknown composition almost immediately upon collection. Sediment sampled from the lake bottom at 2 m was black in color, smelled strongly of hydrogen sulfide, and had a hydrocarbon sheen. A salt crust made deep penetration into the sediment difficult and recovered sediment contained a number of large salt crystals. Primary ions in Patience Lake were Na^+ ^and Cl^- ^at 71.6 and 89.1 milliequivalent percentage of the sum of cations or anions respectively in 1978 [1].

### Freefight, Ingebright, Bitter, and Chappice Lakes

Freefight, Ingebright, and Bitter are located in the southwestern portion of Saskatchewan near the Alberta border while Chappice is just west of the border in Alberta (Fig. 1). Although located within a small geographic area the lakes are quite dissimilar. Freefight is a deep water, permanently stratified lake [33]. Ingebright, Bitter, and Chappice are shallow and ephemeral.

**Figure 3:**
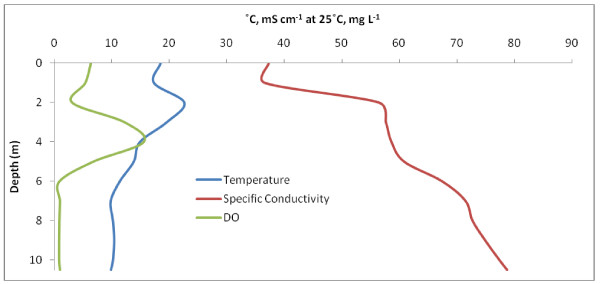
**Freefight Lake**. Freefight depth profiles for temperature (°C), specific conductivity (mS cm^-1 ^at 25°C), and DO (mg L^-1^). Some weak stratification can be seen along with a primary production maximum at 4 m.

Freefight Lake is eusaline within the mixolimnion. Water column chemical and physical properties to a depth of 10.5 m are shown in Fig. 3 and Table 2. Although sampling was limited to this depth, the lake is known to approach a depth of 25 m [33]. Fig. 3 shows a shallow, weak pycnocline at 2 m and a chemocline at 6 m below which suboxic conditions exist. A strong region of primary productivity was identified at 4 m based on a supersaturation of DO. The Secchi depth was determined to be 2.5 m. Sediment recovered from 10.5 m smelled strongly of hydrogen sulfide suggesting that sulfate reduction is occurring. Access to the southern shore of Freefight Lake was found via fenced rangeland.

**Table 2:** Chemical and physical properties for Manito, Redberry, Freefight, Little Manitou East, Little Manito West, and Big Quill Lakes.

Lake	Depth (m)	Temperature (°C)	Specific Conductivity (mS cm^-1 ^at 25°C)	DO (mg L^-1^)	pH
Manito	0	15.78	38.23	7.83	9.53
	0.5	15.77	38.31	7.8	9.53
	1	15.75	38.31	7.69	9.53
	1.5	15.69	38.31	7.59	9.53
	2	15.67	38.31	7.53	9.54
	2.5	15.4	38.31	7.54	9.53
	3	15.67	38.31	7.53	9.53
	3.5	15.1	38.31	7.43	9.53
	4	14.92	38.32	7.3	9.53
	4.5	14.48	38.37	7.09	9.52
	5	14.27	38.41	6.48	9.52
	5.5	13.97	38.53	6.31	9.5
	6	6.12	41.5	1.38	9.43

Redberry	0	15.19	17.68	9.77	8.82
	1	15.18	17.68	9.71	8.83
	2	15.17	17.68	9.74	8.83
	3	15.15	17.68	9.5	8.82
	4	15.08	17.69	9.8	8.83
	5	14.81	17.69	9.66	8.83
	5.5	14.44	17.69	9.78	8.83
	6	14.15	17.69	9.76	8.82
	6.5	14.04	17.76	9.86	8.8
	7	7.18	19.66	14.73	8.73
	7.5	4.13	20.11	15.07	8.78
	8	3.17	20.31	14.4	8.74
	9	2.46	20.43	13.75	8.72
	9.5	2.06	20.5	12.95	8.71
	10	2.02	20.51	12.93	8.7

Little Manitou West	0	16.16	53.25	7.83	8.65
	1.5	16.05	53.25	7.36	8.65
	2.5	15.87	53.3	7.06	8.64
	3	15.42	53.53	6.3	8.62
	3.5	14.51	54.04	5.5	8.6
	4	13.23	63.12	0.31	8.43
	4.3	12.16	64.32	0.5	8.42

Little Manitou East	0	17.49	26.3	8.95	8.76
	0.5	17.58	26.7	8.91	8.76
	1	18.39	51.04	12.98	8.7
	1.5	18.55	53.25	10.18	8.65
	2	17.41	59.1	20.62	8.54

Freefight	0	18.55	37.36	6.43	8.87
	1	17.37	36.67	5.46	8.93
	2	22.66	56.49	3.18	8.77
	3	19.62	57.71	12.24	8.79
	4	14.95	58.68	15.56	8.7
	5	13.87	60.87	6.85	8.58
	6	11.44	67.48	1	8.25
	7	9.86	71.55	1.07	8.06
	8	10.23	72.76	0.99	7.99
	9	10.47	74.96	0.95	7.89
	10	10.24	77.44	0.95	7.81
	10.5	9.9	78.77	1.07	7.77

Big Quill	0	16.03	20.27	6.25	8.71
	1	16.03	20.28	6.25	8.71
	2	15.89	20.29	6.02	8.70
	3.3	15.62	20.44	5.39	8.70

Chappice Lake is a shallow, hypersaline playa [34] located approximately 80 km west of Freefight Lake. Readings taken from just below the surface several meters offshore recorded a DO concentration of 4.82 mg L^-1 ^and pH of 9.09. Access to Chappice was by rangeland to the south of the lake.

Hypersaline Ingebright Lake is the site of a mothballed sodium sulfate extraction plant owned by Saskatchewan Minerals and the largest Na_2_SO_4 _deposit in North America [5]. Measurements were made from a boom between the former evaporation pond and reservoir pond in 1.4 m of water. The current state of the plant allows for a free exchange of water between the evaporation pond and reservoir pond. Conductivity, pH, and DO were constant with depth. Specific conductivity ranged from 97.33 mS cm^-1 ^at 25°C on the surface to 97.39 mS cm^-1 ^at 25°C at 1 m. DO ranged from 1.39 mg L^-1 ^on the surface to 1.30 mg L^-1 ^at depth while pH remained constant at 8.43. The Secchi depth was determined to be 0.75 m. Access to Ingebright was across rangeland to the west of the lake to the point where the evaporation and reservoir ponds exchange.

Bitter Lake is an extensive network of large, hypersaline playas lying on the border between Alberta and Saskatchewan and is adjacent to the playas of Many Island Lake. The exact sampling location is given in Table 1. After the Chaplin Lakes system Bitter Lake hosted the largest abundance of microbial mats of the lakes investigated. Because of its shallow nature *in situ *measurements at Bitter Lake were not possible. Measurements made from a water sample indicated a specific conductivity of 106.2 mS cm^-1 ^at 25°C and pH of 8.79. Sediment recovered in 10 cm of water was black, sandy, firmly packed, and smelled weakly of hydrogen sulfide. Historically the major cation in Bitter Lake was Na^+ ^comprising 23.8% of total cations, while the major anions are Cl^- ^and SO_4 _^2- ^which comprised 38.3% and 60.4% of total anions, respectively [1]. Access to the southern shore of Bitter Lake was made across fenced rangeland from Canada Highway 1.

### Chaplin Lakes

**Figure 4:**
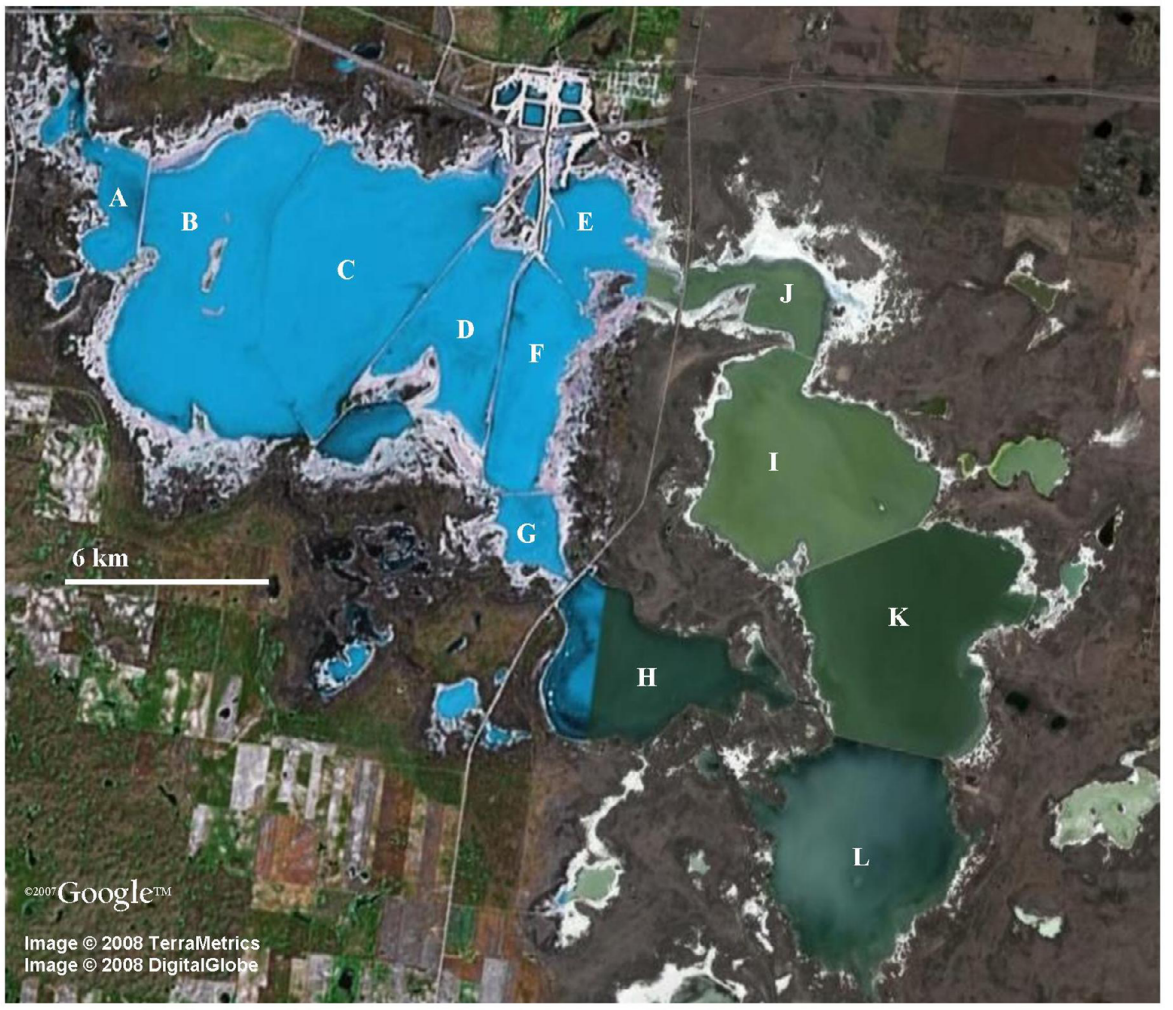
**Satellite image of the Chaplin Lakes**. A. Uren Bay B. West Chaplin Lake West Division #2 C. West Chaplin Lake West Division #1 D. West Chaplin Lake Center Division E. West Chaplin Lake NE Division West Side F. West Chaplin Lake SE Division G. Hughes Bay H. Midtskogen Bay I. East Chaplin Lake North Division North Side J. West Chaplin Lake NE Division East Side K. East Chaplin Lake North Division South Side L. East Chaplin Lake South Division. Printed with the permission of Google Earth.

The Chaplin lakes are located near the town of Chaplin in south central Saskatchewan. Reconstructed from several lakes into a network of solar evaporation ponds by Saskatchewan Minerals for a sodium sulfate extraction facility, the Chaplin lakes represent a wide range of salinities across a small geographic area. The configuration of the evaporation ponds in 2007 is shown in Fig. 4. Water chemistry data for the Chaplin Lakes is shown in Table 3. Ion concentrations for the current Chaplin lakes are not known, however historic data for "Chaplin East" and "Chaplin West" are available [1]. These data indicate that SO_4 _^2- ^was the major anion in both lakes comprising 88% of the total anions in Chaplin West and 90.8% in Chaplin East. In the case of both lakes the major cation was Na^+^, making up 82.6% of the total cations in Chaplin West and 88.7% in Chaplin East [1].

**Table 3:** Chemical and physical properties of the Chaplin Lakes.

Lake	Designation in Fig. 4	Temperature (°C)	Specific Conductivity (mS cm^-1 ^at 25°C)	pH	TDS (g L^-1^)	Absolute Salinity (ppt)	DO (mg L^-1^)
West Chaplin West Division 1	C	20.45	108.5	8.66	235.74	184	2.58
West Chaplin Center Division	D	19.97	99.00	8.99	192.39	155	6.00
West Chaplin SE Division	F	17.11	89.08	8.8	144.82	126	-
West Chaplin NE Division West Side	E	13.31	84.75	8.94	-	-	.96
West Chaplin NE Division East Side	J	16.94	47.35	9.19	60.14	54	5.68
West Chaplin West Division 2	B	18.6	46.62	-	152.31	137	-
Midtskogen	H	19.27	18.35	9.01	-	-	7.75
Hughes Bay	G	21.30	15.60	9.27	-	-	-

**Figure 5:**
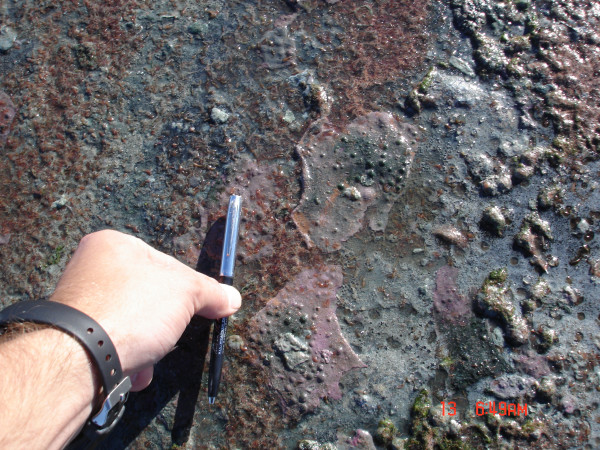
**Microbial mat at West Chaplin Lake NE Division**. All of the Chaplin lakes with a specific conductivity above 40 mS cm^-1 ^at 25°C contained microbial mat communities similar to the one pictured here, from West Chaplin Lake West Division #1.

All of the Chaplin lakes with a specific conductivity above 40 mS cm^-1 ^contained abundant microbial mat communities similar to the one pictured in Fig. 5. Sediment collected from West Chaplin West Division 1, West Chaplin NE Division, West Chaplin Center Division, and West Chaplin SE Division smelled strongly of hydrogen sulfide. Access to the Chaplin Lakes was from Provincial Highway 58 and, with permission, from private causeways controlled by Saskatchewan Minerals.

### Muskiki Lake

Muskiki is a shallow, hypersaline playa northeast of Saskatoon. Maximum depth sounded was 0.75 m. The water column was well mixed; specific conductivity ranged from 57.82 mS cm^-1 ^at 25°C at the surface to 58.06 mS cm^-1 ^at 25°C at depth. DO ranged from 5.74 mg L^-1 ^at the surface to 5.16 mg L^-1 ^at depth, and pH remained constant with depth at 8.59. Sediment recovered from the bottom of the lake was black in color, contained a number of large salt crystals (intermixed within the sediment), and had a strong tar-like odor. Historically, Muskiki had the highest percentage of SO_4 _^2- ^relative to other anions of any lake investigated at 93.5% [1]. The primary cation present is Na^+ ^at 52.6% of total cations [1]. Access to Muskiki was found along the west shore where Provincial Highway 2 approaches the lake.

### Little Manitou East and West

**Figure 6:**
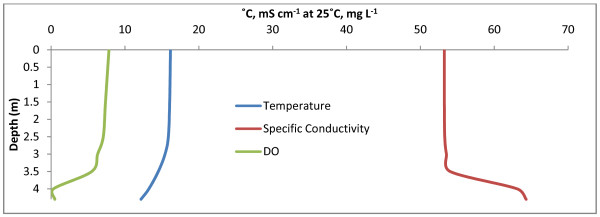
**Little Manitou West**. Depth profile for Little Manitou West with temperature (°C), specific conductivity (mS cm^-1 ^at 25°C), and DO (mg L^-1^). The strong chemocline between 3.5 m and 4 m can be clearly seen.

The Little Manitou system is comprised of two lakes southeast of Saskatoon. The two lakes are separated by a semi-permeable causeway formed by Provincial Highway 365. Stratified and eusaline (within the mixolimnion), Little Manitou West is considerably larger and deeper then Little Manitou East. Measurements were made to a depth of 4.3 m at the location given in Table 1, maximum lake depth is reported as 5.2 m [4]. Water chemistry data is presented in Table 2 and depth profiles of temperature, specific conductivity and DO are shown in Fig. 6. A strong chemocline was observed between 3.5–4 m in which DO dropped from 5.5 to .3 mg L^-1 ^while salinity and specific conductivity increased from 57.82 to 63.12 mS cm^-1 ^at 25°C. A weak thermocline was observed below 2.5 m. Sediment recovered from a depth of 4.3 m consisted of mostly algal material. It was not possible to determine at what depth this algal material originated, however given the anoxic bottom water it was unlikely to have come from below 3.5 m.

**Figure 7:**
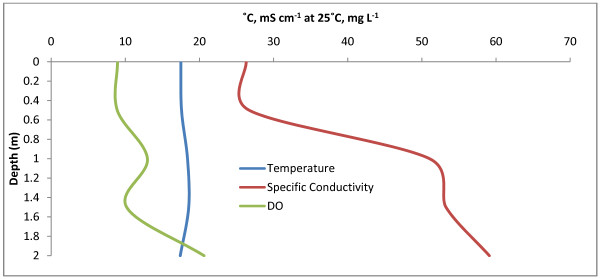
**Little Manitou East**. Depth profile for Little Manitou East with temperature (°C), specific conductivity (mS cm^-1 ^at 25°C), and DO (mg L^-1^). Some correlation between DO and specific conductivity can be seen throughout the water column.

Little Manitou East is a shallow, eusaline lake with a Secchi depth exceeding the 2 m depth of the lake. The turbidity and salinity of Little Manitou East allows for abundant aquatic vegetation and a well oxygenated water column. Water chemistry and physical properties are listed in Table 2. Fig. 7 shows temperature, specific conductivity, and dissolved oxygen profiles for the lake. Temperature remained relatively constant with depth throughout the water column. The oxygen and salinity maxima were on the bottom at 2 m. Sediment recovered from a depth of 2 m was black, with a strong hydrogen sulfide odor and contained some aquatic vegetation. Access to East and West Little Manitou was from the causeway separating the two lakes.

### Manito Lake

**Figure 8:**
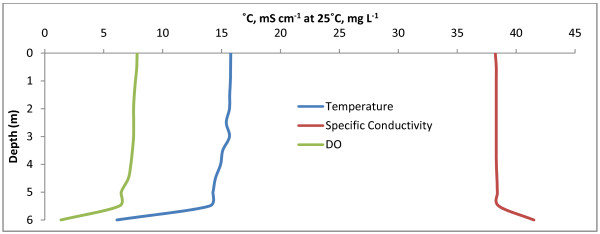
**Manito**. Depth profile for Manito with temperature (°C), specific conductivity (mS cm^-1 ^at 25°C), and DO (mg L^-1^). A strong pycnocline and chemocline can be seen at a depth of 5.5 m.

Manito Lake is a large, deep, stratified, polysaline lake located northwest of Saskatoon near the Alberta border. Data were collected to a depth of 6 m at the location listed in Table 1, maximum lake depth is given as 21.5 m [4]. The Secchi depth was determined to be 1.8 m. Sediment recovered from a depth of 6 m smelled strongly of hydrogen sulfide. Historically the dominant cation in Manito was Na^+ ^at 89.5% of total cations and the dominant anion was SO_4 _^2- ^at 67.1% of total anions [1]. Water chemistry and physical properties in Manito are presented in Table 2. Depth profiles for temperature, dissolved oxygen, and specific conductivity are shown in Fig. 8. Manito Lake exhibited a well mixed surface layer from 0–5 m. At 5.5 m a strong pycnocline and chemocline were found. Access to Manito Lake was from Provincial Highway 40 at the northwest corner of the lake.

### Big Quill Lake

Big Quill is the largest member of a three lake system east of Saskatoon that includes Little Quill Lake and Mud Lake. It is also home to a potassium sulfate production facility and a commercial fishery for *Diaptomus *and *Artemia*. Despite its large surface area the deepest point sounded at Big Quill was 3.3 m, although deeper spots exist. Interviews with commercial fisherman revealed that numerous deep holes can be found in the lake as a result of past use as a bombing range by the Canadian RAF. The Secchi depth was 1 m. Historically the major cation in Big Quill was Na^+ ^at 46.5% of total cations [1]. The predominant anion was SO_4 _^2- ^at 84.4% of total anions [1]. Water chemistry and physical properties are listed in Table 2. The lake was well mixed to the bottom with little variation in the measured parameters. Access to Big Quill was from the southern shore near the Big Quill Resources potassium sulfate plant.

### Redberry Lake

**Figure 9:**
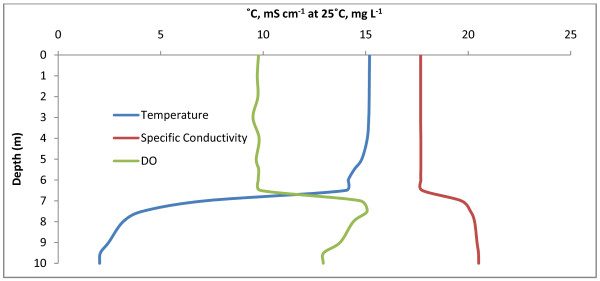
**Redberry**. Depth profile for Redberry Lake with temperature (C), specific conductivity (mS cm^-1 ^at 25°C), and DO (mg L^-1^). DO is seen to parallel specific conductivity down to the oxygen maximum at 7 m.

Redberry is a large mesosaline lake located north of Saskatoon at the Redberry Lake UN Biosphere Reserve. The reserve is a critical habitat for several species of migratory birds, most notably the American white pelican and piping plover [29]. Based on turbidity Redberry was the most oligotrophic lake investigated with a Secchi depth of 4 m. This comparison is consistent with observations made by Waiser and Roberts [35]. Measurements were made in 10 m of water, maximum lake depth is 17 m [35]. Water chemistry data for Redberry are presented in Table 2 and the depth profiles of temperature, specific conductivity, and DO with depth are shown in Fig. 9. This figure shows a strong parallel between specific conductivity and dissolved oxygen down to the oxygen maximum at 7.5 m. Both a strong pycnocline and chemocline were present at this depth. Sediment from 10 m was grey with a green and cream colored covering and did not smell of hydrogen sulfide. Historic ion concentrations in Redberry Lake are 93.1% of total anions for SO_4 _^2- ^and 67.4% of total cations for Mg^2+ ^[1]. Redberry was the only lake investigated where Mg^2+ ^was the dominant cation. Access to Redberry Lake was through the park entrance on the west side of the lake.

## Discussion

### Long term salinity trends

**Figure 10:**
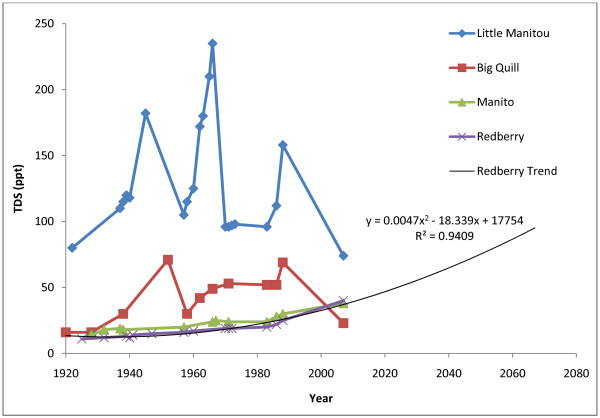
**Extension of Hammer time series**. Data for all lakes from 1920 to 1988 [32]. Some data for the period 1990–2007 is available but has not been used due to reporting in g L^-1 ^or specific conductivity [7, 43]. TDS has decreased slightly since 1971 for Little Manitou West. A sharper decrease can be seen for Big Quill Lake, although the lake is still above historic minimums. Redberry and Manito Lakes have both increased in TDS since 1971.

Fig. 10 shows the continuation of a time series compiled by Hammer [1] for Little Manitou West, Big Quill, Redberry, and Manito. The change in TDS for Redberry can be described by a second order polynomial with an R^2 ^value of 0.94. A projection of this trend to 100 ppt is displayed. TDS (in ppt) decreased from 1971 to the present in Little Manitou and Big Quill, and increased in Redberry and Manito. In the case of Little Manitou the mechanism of this decrease is known to be the diversion of water into the lake from the South Saskatchewan River [1]. The start of this diversion coincides with the 1968 salinity maximum shown in Fig. 10.

The slow decrease in salinity at Big Quill can also be attributed to water management. Historic lows for TDS in Big Quill are near 15 ppt [32]. Placement of a dam between Big Quill and Little Quill in 1936 initiates a period of widely varying salinity [32]. During times of high precipitation water is allowed to flow into Big Quill from the Little Quill Drainage, decreasing salinity. During times of low precipitation water is retained in Little Quill [19]. Both of these measures are augmented by the large surface area to volume ratio (307.4 km^2^:449 × 10^6 ^m^3^) of Big Quill that amplifies the response of salinity to both precipitation and evaporation [4]. The potential economic effects of decreasing salinity in Big Quill Lake cannot be overlooked. Both the commercial fishery and potassium sulfate production facility require a certain minimum concentration of salt. This makes understanding the mechanisms controlling salinity in Big Quill a priority and water managers should be aware of the minimum values necessary for commercial and ecological viability.

Water management projects do not involve the Redberry or Manito lakes directly, and the salinity of these lakes can be expected to respond naturally to evaporation and precipitation with some effects from aquifer depletion or perforation [36]. Currently evaporation exceeds precipitation in most North American prairie regions, leading to the expectation of increasing salinities over time [16,32,36-38]. A closer look at any particular basin will require local long term precipitation records. Unfortunately, long term precipitation records are not available for Manito Lake; climate data for the nearby station in Lloydminster is only available from 1983 on [39]. However such records are available for a weather station 48 km south of Redberry Lake near the city of Saskatoon [39].

**Figure 11:**
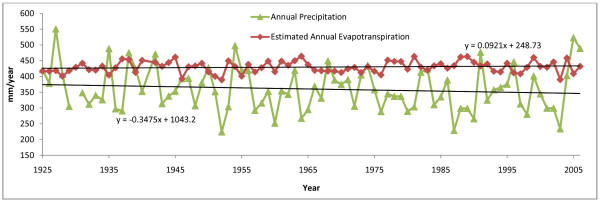
**Annual precipitation and evapotranspiration for Redberry Lake**. Relatively constant rates of precipitation and evapotranspiration (calculated with a modified Thornwaite equation [40]) are shown for Redberry Lake. This suggests a mechanism other than climate for the nonlinear increase in salinity at Redberry.

Fig. 11 shows annual total precipitation (rain and snow) from 1925 to 2006 for the Saskatoon weather station. Data for the years 1930 and 1941 are not available. A linear trend line for these annual precipitation totals has a slope of -0.347 mm yr^-1^, indicating that precipitation totals have remained relatively constant since 1930. The Redberry Lake catchment is estimated to be 1180 km^2 ^[29]. For a drainage this size a deficit of 0.347 mm of water in a given year results in 413 m^3 ^less water available to dilute the terminal basin every year, a small fraction of the estimated 1999 lake volume of 2.53 × 10^8 ^m^3 ^(1.63 × 10^-6 ^% of lake volume). Fig. 11 also shows annual estimated evapotranspiration determined by a modified Thornwaite Equation [40]. This equation does not take into account the strong evaporative effects of wind direction and speed in a prairie environment. However diurnal and annual variations in wind direction and speed have remained constant since they were first recorded in 1957 [39]. The steady average rate of evapotranspiration suggests that the nonlinear increase in salinity shown in Fig. 10 does not result from a decadal-scale shift in climate. Another factor, such as basin morphology may be responsible.

**Figure 12:**
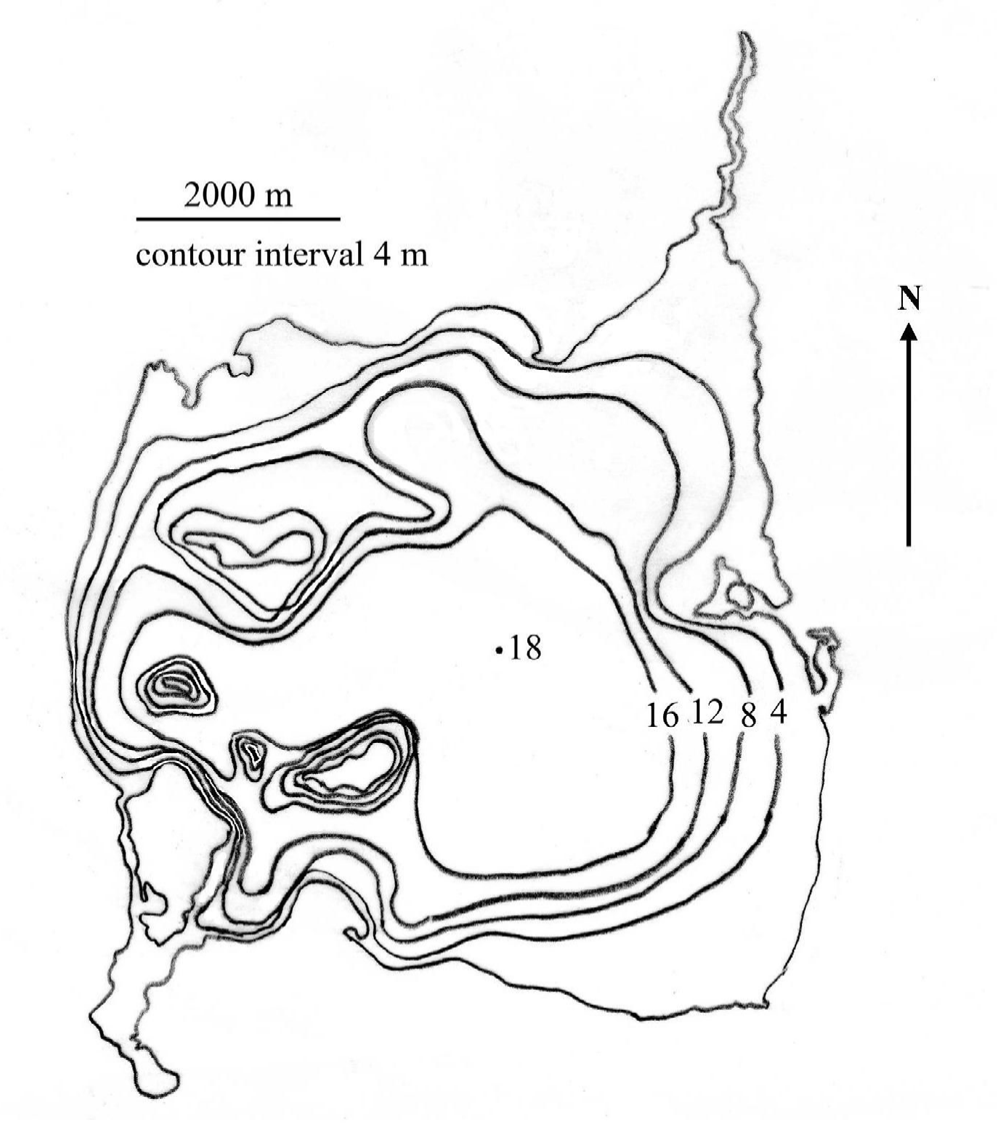
**Redberry Lake bathymetry**. This illustration of the bathymetry for Redberry Lake is redrawn from Hammer [4]. The roughly conical shape of the basin can be clearly seen. As the lake level drops, surface area increases relative to volume. This increases evaporation relative to volume, resulting in a nonlinear increase in salinity.

**Figure 13:**
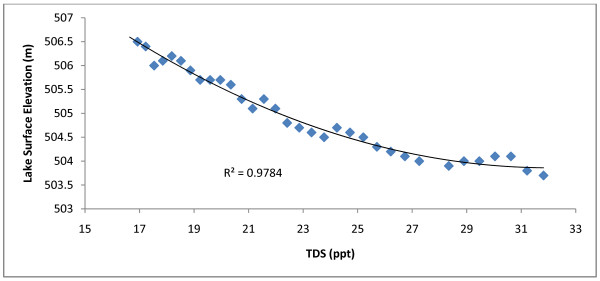
**TDS vs. lake surface elevation at Redberry Lake**. TDS was derived by the equation displayed in Fig. 10. Derived TDS are shown to correlate well with surface elevation [29] at Redberry Lake. This correlation reflects the increasing rate of salinity change at Redberry.

Redberry Lake possesses a roughly conical basin morphology, as illustrated by Hammer in Fig. 12[4]. As the surface area to volume ratio of Redberry Lake increases with decreasing lake depth, the fraction of the lake which evaporates each year will increase in a nonlinear manner. Fig. 13 illustrates this effect. Annual TDS for Redberry Lake are determined as a function of time according to the equation shown in Fig. 10. The close correlation between lake surface elevation above sea level [29] and TDS (which increases inverse to the decrease in volume) can be seen along with a nonlinear decrease in the ratio of lake elevation to TDS.

Due to the ecological significance of the lake increasing salinity at Redberry is cause for concern, just as the decreasing salinity at Big Quill is cause for economic concern. If the current trend for Redberry Lake continues, salinity may reach 100 ppt by 2070 as shown in Fig. 10. The effects of rising salinity have already been seen on several native and introduced species of fish at Redberry Lake. Reports from the 1920s indicate that northern pike inhabited Redberry Lake, a testament to the low salinity at that time [29]. Lake whitefish, walleye, and rainbow trout were all stocked in the mid-20^th ^century, but no population has persisted. Several small species of marine fish remain at Redberry including the brook stickleback or *Culaea inconstance *[29]. Salinity at Redberry is already exceeding values typically found in marine environments and it is unlikely that any fish species will remain far into the future.

The effect of rising salinity at Redberry and other prairie lakes on invertebrate species is less clear. It has been demonstrated that high salinity does not necessarily correlate with low productivity [14,16,23], though there is some disagreement as to whether biomass declines as salinity increases [6,23]. There is good evidence that phytoplankton and invertebrate species richness declines with rising salinity [6,13,23,25,27]. What effect this loss of prey diversity will have on migratory bird populations at Redberry Lake remains to be seen. Reduced food availability due to rising salinity is cited as a likely reason for the delayed nesting of white winged scoters at Redberry [30].

### The impact of salinity on other parameters

Salinity does not appear to be a determining factor for other chemical characteristics. No correlation was observed between TDS and dissolved oxygen, pH, or specific conductivity. Although pH is controlled in part by the concentration of Ca^+ ^and CO_3 _^2-^, these ions are thought to be present in low concentrations compared to the major ions Na^+^, SO_4 _^2-^, K^+^, and Cl^- ^[1]. This differentiates these lakes from the "soda" lakes common in other hypersaline environments where Ca^+ ^and CO_3 _^2- ^make up a significant portion of the ions present. pH is probably more strongly controlled by the production and utilization of CO_2 _within the water column [5].

Finally, no correlation was observed between specific conductivity and TDS. This highlights a fundamental problem in investigating athalassohaline lakes; the widely used practice of determining salinity as a function of specific conductivity does not work when the anion charge to ion ratio is not 1:1 [41]. The dominant anion within the Great Plains of Western Canada's lakes is almost always SO_4 _^2-^, but it is never the only anion present in high concentrations. This makes specific conductivity useful only as a rough guide to the salinity of a specific lake (high specific conductivity will always correlate with high TDS but not by any set conversion factor), or as an indicator of change within a single lake. Despite its limitations specific conductivity has been included here to provide a means of comparison with other sources.

## Conclusion

The saline lakes of Canada's Great Plains provide a suitable testing ground for salinity proxies. Relatively easy access is available to lakes ranging in TDS from below 23 ppt to 184 ppt. In addition the high sulfate, low calcium carbonate nature of many of the lakes represents a unique environment.

Salinity is increasing at Redberry and Manito Lakes but is decreasing at Little Manitou and Big Quill Lakes. Long term variations in the salinity of other lakes in the region remain unknown. It is necessary to develop an understanding of the mechanisms behind these changes in order to predict future salinity trends. The economic and ecological importance of many of these lakes makes such an investigation a priority. It is known that the decreasing salinity in Little Manitou and Big Quill lakes are caused by water management projects. The nonlinear increase in salinity at Redberry and Manito Lakes may result from basin morphometry combined with a negative water balance during the 20^th ^century.

## Methods

During June 2007, 19 lakes were investigated throughout the Northern Great Plains region of Canada. Dissolved oxygen, specific conductivity, water temperature, and pH were measured with an YSI-556 multiprobe. Where shore conditions and lake depth permitted, readings were taken by boat from the point at which lake depth began to stabilize as determined by a Hawkeye Digital Sonar handheld sonar system. Where it was not possible or practical to deploy the YSI-556 multiprobe by boat, it was deployed several meters from shore by wading into the lake and/or throwing the probe into deeper water. In some cases it was necessary to collect water in a graduated cylinder before a reading could be made. In these cases DO values are not reported. At the highest salinity lakes serial dilutions were made to insure accurate readings of specific conductivity. In all cases serial dilutions verified the initial readings. When the boat was deployed the Secchi depth was determined by lowering a Secchi disk on a marked rope to the depth from which it could not be distinguished from the surrounding water without the aid of polarizing glasses.

Filtered and unfiltered water samples were collected *in situ *using a Masterflex peristaltic pump to draw water from depth. Water depths reported as 0 m indicated that water was drawn from just below the surface. Samples were filtered using 142 mm Pall-Gelman A/E glass fiber filters with a 1.0 μm pore diameter. Filtered and unfiltered water samples were collected in PET1 plastic bottles sealed with electrical tape to minimize evaporation. The high alkalinity present at many of the sampled lakes caused deterioration of the PET1 bottles necessitating a transfer of the samples to LDPE bottles within 3–5 days. Sediment samples were collected either with a stainless steel Van Veen sediment grab from the boat or with a trowel from shore.

TDS were determined in the laboratory using a variation on the procedure for determining filterable residue described by the EPA's Office of Research and Development [42]. Due to the high concentration of salts present a smaller aliquot was used then is recommended by this procedure. In addition a lower initial heat was used to minimize the "popping" and resulting loss of material caused by faster, hotter evaporations. Three 10 ml aliquots of filtered water were transferred by pipette into pre-weighed aluminum weighing pans. The specific gravity of each aliquot was determined by weighing the pan and aliquot on a precision scale, then subtracting the weight of the pan. Pans were evaporated at 60°C for 20 hours, then at 180°C for two hours at which time a stable weight was reached, indicating that all water had been driven from the hydrated salts. Residues were cooled in a desiccator prior to reweighing. Care was taken to insure minimum time outside of the desiccator for each sample. TDS is reported as the average weight of the three measurements and reported in g L^-1^. To eliminate small errors in measuring the initial 10 mL aliquot, salinity is also reported as absolute salinity following the guidelines presented in Anati [41]. This index provides the most accurate salinity measure for comparison with future work and across lakes with varying ionic compositions.

An effort was made to measure suspended particle concentrations in the lab from unfiltered water collected in the field. A 50 mL aliquot of water was measured in a volumetric flask then filtered through a pre-weighed 47 mm Pall membrane filter with a pore diameter of 0.45 μm. For samples of high salinity the filter was rinsed briefly with distilled water. Filters were then dried at 60°C for two hours at which time a constant weight was reached. Filters were cooled in a desiccator then weighed on a precision scale. The suspended particle concentration was determined as the weight of the filter and particles minus the weight of the filter converted to g L^-1^. A second set of TDS values was determined from the filtrate produced in this procedure. The same technique was used as that already described with the exception that only one 50 ml aliquot was evaporated for each sample. Results from this effort suggest that it is not possible to separate particulate organic matter (POM) from inorganic precipitates with a high degree of accuracy or precision by membrane filtration.

## Competing interests

The authors declare that they have no competing interests.

## Authors' contributions

JB drafted the manuscript, participated in the design and execution of the field work, and conducted analyses of TDS and suspended particles. JS devised the project, helped to draft and edit the manuscript, directed the field team, and oversaw all aspects of data collection. All authors read and approved the final manuscript.
